# Comparative Analysis of Nutritional Components and Phytochemical Attributes of Selected Thai Rice Bran

**DOI:** 10.3389/fnut.2022.833730

**Published:** 2022-02-24

**Authors:** Jiratchaya Wisetkomolmat, Chaiwat Arjin, Apinya Satsook, Mintra Seel-audom, Warintorn Ruksiriwanich, Chanakan Prom-u-Thai, Korawan Sringarm

**Affiliations:** ^1^Department of Animal and Aquatic Sciences, Faculty of Agriculture, Chiang Mai University, Chiang Mai, Thailand; ^2^Department of Pharmaceutical Sciences, Faculty of Pharmacy, Chiang Mai University, Chiang Mai, Thailand; ^3^Lanna Rice Research Center, Chiang Mai University, Chiang Mai, Thailand; ^4^Cluster of Research and Development of Pharmaceutical and Natural Products Innovation for Human or Animal, Chiang Mai University, Chiang Mai, Thailand; ^5^Division of Agronomy, Department of Plant and Soil Sciences, Faculty of Agriculture, Chiang Mai University, Chiang Mai, Thailand

**Keywords:** anthocyanins, nutritional quality, phenolic compound, rice bran, vitamin E, Gamma Oryzanol

## Abstract

It is important to raise awareness regarding rice's nutritional quality and health benefits in terms of enhancing rice consumption in people's daily diets. This study evaluated the proximate components and phytochemical profiles of 11 Thai rice bran varieties, 4 non-colored rice brans and 7 colored rice brans, collected from the same agricultural fields. The chemical composition (ash, fat, proteins, fiber, and gross energy) was determined using proximate analysis methods. High-performance liquid chromatography was used to analyze the γ-oryzanol, tocopherols, and anthocyanins, while gas chromatography mass spectrometry determined the free fatty compounds. The phenolic profile was determined using liquid chromatography-mass spectrometry. The results showed great variations in each parameter of the nutritional and bioactive components among different rice bran varieties. Statistical analysis was also performed correlating the results obtained from PCA to categorize the samples by their nutritional characteristics into three main groups: group A with a high content of protein and fiber, group B with a high content of fat and gross energy, and group C with low fat and energy values but high amounts of functional, active components, particularly γ-oryzanol. Anthocyanins were detected in only one sample of colored rice bran. The major free fatty acids found in rice bran samples were oleic, linoleic, and palmitic acids. Systematic assessment of the concentration of these compounds gained from this study would be helpful to the industrial sector for selecting phytochemical-rich varieties as a value-added component in nutritional products.

## Introduction

Rice (*Oryza sativa* L.) is one of the most widely consumed staple foods worldwide. The caryopsis can be differentiated based on its colors such as brown, white (polished brown rice), red, purple, or black. On a global level, around 755 million tons of rice were produced from 2019 to 2020, which is equivalent to 504 million tons of milled rice (1). Thailand is recognized as one of the centers of origin of rice diversity in the world (2, 3); more than 5,000 varieties of rice are known within the area (4) and the country devotes 66% of its agricultural land to rice production (5, 6).

At present, special varieties of rice are being developed that respond to the needs of consumers in Thailand. As a result, a variety of rice has developed that benefits in a range of fields with varying nutrient values. For instance, the purple hull of Kum Doi Saket, a glutinous purple rice variety, is rich in flavonoids, especially anthocyanins, compared to other purple rice varieties (7). Meanwhile, the non-glutinous white rice, Kao Dok Mali 105, is a unique rice variety with a particular texture and aroma; it has a smooth texture and a natural fragrant aroma of 2-Acetyl-1-pyrroline when cooked (8).

Rice production results in three major parts: the husk, straw, and bran. In the process of rice milling, rice bran is a large and underutilized product of these activities, constituting about 8–10% of the rice grain (9, 10). Consequently, more than 80 million tons of bran are the by-product of rice milling worldwide (11). The bran fraction is defined as the extent to which the germ and bran layers of the brown rice kernel have been removed during the polishing process to yield white rice (9, 12). It contains phenolics, γ-oryzanol, tocopherols, and tocotrienols, along with other health-beneficial nutraceutical components (13–15). In milled rice, protein (6–7%) is the second most prevalent element, carbohydrates accounting for around 78% of the total. Rice bran, on the other hand, has a high protein content (11–15%) as well as being an excellent source of fiber (7–11%). Furthermore, rice bran contains significantly more nutrients and vitamins than the other rice milling fractions (16). Rice bran contains approximately 20% lipids. Rice bran oil has attracted attention for its unique nutraceutical properties and fatty acid content (17). It is, however, bran that has traditionally been used as animal feed but is undervalued as a human food source (18, 19).

Rice bran has been studied for its potential biological functions in recent years viz. anti-inflammatory, antimutagenic, anticarcinogenic, antibacterial, and antioxidant properties (20). These functional properties of rice bran indicate that it is ideal for commercial uses in the food and beverage industry, as well as the nutraceutical and pharmaceutical industries (11, 13, 21). Since rice bran is gaining traction as a source of health-promoting nutraceuticals and novel varieties of these raw materials have been developed, it is worthwhile investigating the profile of Thai rice bran varieties with special grain qualities for functional uses.

Therefore, the objective of this study was to evaluate the physicochemical properties, fatty acid compositions, and content of bioactive compounds (anthocyanins, γ-oryzanol, tocopherols, and phenolic compounds) of raw materials, especially among those qualified special quality rice bran. Moreover, the relationship of the nutraceuticals in each rice bran was investigated using multivariate analysis. The research outcome will be relevant for future research and applications of each rice bran component in the production of functional foods or related items as value-added ingredients.

## Materials and Methods

### Plant Materials

Eleven rice bran samples were provided by Lanna Rice Research Center, Chiang Mai University, Chiang Mai, Thailand. The rice varieties were grown in the demonstration field with the same conditions and practices in the wet season during June–December in 2020. The samples were harvested at maturity before being processed: husking to remove the seed coats from brown rice and polishing to separate the white rice and bran fraction, respectively. The screw press method was used to remove the oil from the rice bran in order to obtain the defatted raw material. After removal of the oil, defatted rice bran was used for the analysis of bioactive compounds. The bran samples were kept at −20°C in sealed bags until further analysis (Table 1).

**Table 1:** Description of rice varieties with special grain quality used in the examination.

**Variety name**	**Abbreviation**	**Pericarp color**	**Endosperm type**	**Special quality**
Khao Dawk Mali 105	KDML 105	Non-colored	Non-glutinous rice	Aroma
Buebang 3 CMU	BB 3 CMU	Non-colored	Non-glutinous rice	Vitamin E
Buebang 4 CMU	BB 4 CMU	Non-colored	Non-glutinous rice	Zinc
Rice Department 6	RD 6	Non-colored	Glutinous rice	Aroma
Kum Chao Morchor 107	KC CMU 107	Purple color	Non-glutinous rice	Antioxidant
Bien Koo 5 CMU	BKU 5 CMU	Purple color	Non-glutinous rice	Iron
K 4 CMU	K 4 CMU	Purple color	Non-glutinous rice	Antioxidant
Kum Doi Saket	KDK	Purple color	Glutinous rice	Gamma Oryzanol
Kum Akha 1 CMU	KAK 1 CMU	Purple color	Glutinous rice	Antioxidant
Sang 5 CMU	Sang 5 CMU	Purple color	Glutinous rice	Phenol
Pieisu 1 CMU	PES 1 CMU	Purple color	Glutinous rice	Anthocyanin

### Chemical Reagents and Standards

All the standards used were purchased from Sigma-Aldrich (Germany). These included standards of tocopherols (α-, β-, γ-, and δ-tocopherol), polyphenols (gallic acid, (+)-catechin, chlorogenic acid, caffeic acid, (-)-epicatechin, (-)-epigallocatechin gallate, 4-hydroxybenzoic acid, *o*-coumaric acid, *p*-coumaric acid, phytic acid, quercetin, rosmarinic acid, and rutin) and anthocyanins (cyanin-3,5-diglucoside, cyanidin chloride, cyanidin-3,5-diglucoside and peonidin-3-glucoside). Dichloromethane, methanol, hexane, and isopropanol (HPLC grade) were purchased from Merck (Darmstadt, Germany). Ethanol was obtained from RCI Labscan Limited (Bangkok, Thailand). Fatty acid methyl ester standard mixtures were purchased from Restek Corporation (PA, USA). Other reagents and chemicals used in the experiments were of analytical grade.

### Chemical Composition and Mineral Analyses

The proximate composition of rice bran varieties, including dry matter content, ash content, crude fiber, crude protein, crude fat, gross energy, and mineral content, was determined following the methods of the Association of Official Analytical Chemists (22). Briefly, moisture content was estimated following method 934.01, ash content was determined by burning in a muffle furnace at 550°C (method 942.05), and crude fiber was determined following method 962.09. The crude protein was determined by the Kjeldahl procedure and calculated as nitrogen × 6.25 (method 2001.11). Crude fat was determined using Soxhlet extraction (Soxtec™ 8000, FOSS, Denmark) (method 2003.06). Gross energy was analyzed in duplicate using an oxygen bomb calorimeter (AC500 Isoperibol Calorimeter for Gross Calorific Content; LECO, St. Joseph, MI, USA). Levels of minerals were determined using an Agilent 240 FS flame atomic absorption spectrophotometer (Agilent Technologies, USA) after calibrating the equipment with respective standard solutions according to the official method prescribed by the Association of Official Analytical Chemists.

### γ-Oryzanol Analysis

Soxhlet extraction was performed to extract the oil from rice bran samples (22). The oils were dissolved with dichloromethane at a final concentration of 1 mg/ml then filtered through a 0.45 μm syringe filter. The samples were then analyzed using a Shimadzu UV-Vis detector with a diode array detector (DAD) (SPD-20A; Shimadzu, Kyoto, Japan) and an Ultra C18 column (5 μm, 4.6 × 250 mm; Restek, PA, USA). The mobile phase was methanol, acetonitrile, dichloromethane, and acetic acid (50:44:3:3) with a flow rate of 1.4 mL/min. The UV detector was set at a wavelength of 330 nm (23).

### Analysis of Tocopherols

Tocopherol analysis was modified from the method of Kramer et al. (24) and Arribas et al. (25) by using a Shimadzu HPLC equipped with a fluorescence detector (RF-20A; Shimadzu Corporation, Kyoto, Japan). Tocopherols were separated on a normal-phase Inertsil SIL-100A column (5 μm, 4.6 × 250 mm; GL Sciences Inc., Tokyo, Japan); 0.6% propan-2-ol in hexane was used as the mobile phase for 30 min at a flow rate of 1 mL/min. The fluorescence detector excitation wavelength was 298 nm and the emission wavelength was 325 nm. Quantification was performed by comparison to the fluorescence signal obtained from standards (α-, β-, γ-, and δ-isoforms) of each compound.

### Analysis of Anthocyanins

Both extraction and quantification of rice bran anthocyanins were adapted from the method of Ooe et al. (26). Briefly, the raw materials (20 g) were defatted twice with 200 ml of dichloromethane and dried. The defatted samples were extracted with 20 ml of 0.5% TFA in 95% ethanol, mixed for 30 min to extract anthocyanins, and filtered through a Whatman No. 1 filter paper. The extracts were kept at a temperature of 4°C away from light. The extract samples were filtered through a 0.45 μm filter. Anthocyanins were determined by a HPLC-DAD system (SPD-20A; Shimadzu, Kyoto, Japan) equipped with an Allure C18 column (5 μm, 4.6 × 250 mm; Restek, PA, USA). The mobile phase A was composed of 0.5% TFA in water; mobile phase B was 0.1% TFA in methanol. The gradient was as follows: 0 min, 92% A, 8% B; 50 min, 85% A, 15% B; 60 min, 70% A, 30% B; 65 min, 40% A, 60% B; 75 min, 40% A, 60% B. The flow rate was set at 1 mL/min. The injection volume for the extract was 20 μL. The wavelength for detection of anthocyanin was 280 nm. Standard calibration curves were plotted using the average value of peak areas for triplicate determinations of cyanidin-3,5-diglucoside and peonidin-3-glucoside standards.

### Analysis of Free Fatty Acids

The samples were initially methylated to fatty acid methyl esters, according to Morrisson and Smith (27). The final fatty acid methyl esters were used to determine free fatty acids following the method of Souphannavong et al. (28) and Arjin et al. (29) using a gas chromatography (GC-2030; Shimadzu, Kyoto, Japan) equipped with a 0.25 mm × 100 m × 0.25 μm wall-coated fused wax capillary column (RT-2560; Restek, Bellefonte, PA, USA). Helium was used as the carrier gas. The oven temperature was increased from 100°C and held for 4 min, then increased from 100 to 240°C at a rate of 3°C/min, and then held at 240°C for 20 min. The injector volume was 1 mL, and the flame ionization detector temperature was 250°C. Chromatograms were processed using Lab Solutions (Shimadzu, Kyoto, Japan). The fatty acid composition was obtained by comparison of the peak retention times with the respective fatty acid standards.

### Analysis of Polyphenol Compounds

The samples were extracted with ethanol/water (95:5, v/v) according to the method of Arribas et al. (25). The ethanolic fraction of the extracts was evaporated under reduced pressure (40°C) in order to obtain a residue by using a rotary evaporator (Hei-VAP Precision, Heidolph, Germany). Then, the phenolic compounds were analyzed by applying the method of Mighri et al. (30). Analytical liquid chromatography was performed using an Agilent 1260 Infinity II series chromatogragh, coupled with an Agilent 6130 electrospray ionization quadrupole mass spectrometer (Agilent Tech., Santa Clara, CA, USA). Separation was executed using an Ultra C18 column (5 μm 4.6 × 250 mm; Restek, Bellefonte, PA, USA). The mobile phase was composed of A (0.2% acetic acid in 95% water and 5% MeOH) and B (0.2% acetic acid in 50% water and 50% acetonitrile) with a linear elution gradient: 0–45 min, 10–20% B; 45–85 min, 20–55% B; 85–97 min, 55–100% B; 97–110 min, 100% B; the initial conditions were held for 10 min as a re-equilibration step. The flow rate of the mobile phase was 0.5 mL/min, the column temperature was maintained at 40°C, and the injection volume was 20 μL. Spectra were obtained in the negative selected ion monitoring mode and processed using OpenLab software. High purity nitrogen was used as a nebulizer and auxiliary gas. The mass spectrometer was operated in negative ion mode with a capillary voltage of −3.5 V, a nebulizing gas flow of 1.5 L/min, a dry gas flow rate of 12 L/min, a dissolving line temperature of 250°C, a voltage detector of 1.35 V and the full scan spectra from 100 to 1,200 m/z with 250 ms/spectrum.

### Statistical Analysis

The data for the physical characterization were obtained in duplicate for each sample and expressed as the mean ± standard deviation (SD). The chemical data are presented as mean values ± SD and were analyzed using the *t*-test. All statistical analyses were performed using SPSS software (version 20.0) for analysis of variance (ANOVA). Multiple comparisons by Duncan's Multiple Range Test were used for varietal differences at *P* < 0.05 level. Principal Component Analysis (PCA) was used to investigate the relationship between biochemical parameters using Minitab software.

## Results

### Proximate and Mineral Composition

The proximate and mineral composition of 11 rice bran samples locally grown in Northern Thailand is shown in Table 2. There were significant differences in the proximate and mineral composition of rice bran samples. The ash content was highest in RD 6 (10.27%). The highest percentage of crude fiber among all the rice bran samples was exhibited in BB 3 CMU (6.72%). The crude protein content of glutinous and non-glutinous rice brans was significantly different between samples. The highest protein content was found in purple rice, PES 1 CMU (15.20%) followed by Sang 5 CMU (14.92%) and BKU 5 CMU (14.19%). The sample of BB 3 CMU white rice had the highest fat content of 18.68%, followed by BB 4 CMU (18.55%) and RD 6 (18.42%). Gross energy values were appreciably different among rice bran samples; the highest was found in BB 4 CMU white tice (4,750.55 cal/g sample), followed by BB 3 CMU (4,681.05 cal/g sample) and RD 6 (4,628.25 cal/g sample).

**Table 2:** Proximate and mineral content of rice bran samples.

**Pericarp color**	**Non-colored**				**Purple color**						
**Endosperm type**	**Non-glutinous rice**			**Glutinous**	**Non-glutinous rice**			**Glutinous rice**			
**Varieties**	**KDML 105**	**BB 3 CMU**	**BB 4 CMU**	**RD 6**	**KC CMU 107**	**BKU 5 CMU**	**K 4 CMU**	**KAK 1 CMU**	**Sang 5 CMU**	**PES 1 CMU**	**KDK**
**Proximate composition (%)**											
Dry matter	90.46 ± 0.30^de^	91.53 ± 0.04^a^	91.21 ± 0.00^ab^	90.52 ± 0.17^de^	89.19 ± 0.05^f^	90.86 ± 0.10^bcd^	90.82 ± 0.35^bcd^	91.43 ± 0.23^a^	91.13 ± 0.18^abc^	90.78 ± 0.15^cd^	90.18 ± 0.08^e^
Ash	9.35 ± 0.21^b^	8.22 ± 0.27^d^	8.75 ± 0.08^c^	10.27 ± 0.06^a^	6.19 ± 0.24^f^	7.77 ± 0.56^d^	7.93 ± 0.04^d^	7.12 ± 0.04^e^	8.01 ± 0.02^d^	10.05 ± 0.23^a^	6.73 ± 0.10^e^
Crude fiber	5.26 ± 0.08^d^	6.72 ± 0.09^a^	6.10 ± 0.27^ab^	5.11 ± 0.19^d^	5.47 ± 0.32^cd^	6.14 ± 0.18^ab^	6.20 ± 0.19^ab^	6.00 ± 0.36^bc^	5.05 ± 0.47^d^	6.48 ± 0.37^ab^	4.29 ± 0.00^e^
Crude protein	12.82 ± 0.15^de^	12.50 ± 0.08^ef^	13.06 ± 0.06^cd^	13.37 ± 0.36^c^	12.04 ± 0.21^f^	14.19 ± 0.29^b^	13.40 ± 0.44^c^	14.00 ± 0.10^b^	14.93 ± 0.10^a^	15.20 ± 0.02^a^	12.61 ± 0.05^de^
Crude fat	16.48 ± 0.19^b^	18.68 ± 0.11^a^	18.55 ± 0.19^a^	18.42 ± 0.40^a^	12.03 ± 0.30^e^	13.76 ± 0.10^c^	16.48 ± 0.62^b^	15.96 ± 0.17^b^	13.48 ± 0.56^cd^	15.83 ± 0.02^b^	12.84 ± 0.59^d^
Gross energy, cal/g	4590.40 ± 60.81^c^	4681.05 ± 43.35^ab^	4750.55 ± 16.62^a^	4628.25 ± 61.73^bc^	4239.35 ± 14.50^f^	4553.05 ± 45.75^c^	4595.00 ± 37.34^c^	4558.95 ± 3.04^c^	4461.95 ± 27.22^d^	4547.00 ± 22.20^c^	4343.20 ± 5.23^e^
**Mineral content**											
K (g/100g)	2.09 ± 0.03^c^	1.98 ± 0.00^e^	2.13 ± 0.00^b^	2.05 ± 0.01^d^	1.31 ± 0.03^i^	1.92 ± 0.01^f^	1.66 ± 0.01^g^	2.02 ± 0.01^d^	1.92 ± 0.01^f^	2.27 ± 0.01^a^	1.42 ± 0.03^h^
P (g/100g)	1.30 ± 0.06^a^	1.19 ± 0.00^cd^	1.25 ± 0.11^abc^	1.29 ± 0.02^ab^	0.99 ± 0.06^e^	1.20 ± 0.01^cd^	1.19 ± 0.01^d^	1.21 ± 0.00^bcd^	1.29 ± 0.02^ab^	1.24 ± 0.02^abc^	1.15 ± 0.04^cd^
Mg (g/100g)	0.76 ± 0.02^b^	0.63 ± 0.00^e^	0.72 ± 0.02^c^	0.82 ± 0.01^a^	0.46 ± 0.01^h^	0.56 ± 0.00^f^	0.62 ± 0.01^e^	0.52 ± 0.01^g^	0.53 ± 0.01^g^	0.69 ± 0.01^d^	0.47 ± 0.02^h^
Ca (mg/kg)	271.75 ± 2.12^bcd^	288.93 ± 35.96^bc^	310.63 ± 13.26^b^	397.18 ± 18.99^a^	266.26 ± 20.88^bcd^	237.03 ± 23.02^de^	376.15 ± 15.98^a^	198.68 ± 21.60^e^	249.86 ± 22.15^cd^	262.48 ± 3.64^cd^	227.54 ± 12.57^de^
Fe (mg/kg)	46.24 ± 0.55^g^	71.62 ± 0.74^c^	83.17 ± 1.09^a^	45.98 ± 0.93^g^	32.94 ± 0.84^i^	64.97 ± 2.01^e^	50.77 ± 0.88^f^	69.02 ± 0.54^d^	65.27 ± 1.09^e^	78.79 ± 1.34^b^	37.01 ± 1.17^h^
Mn (mg/kg)	90.26 ± 2.17^e^	112.88 ± 5.76^cd^	136.50 ± 0.60^b^	121.31 ± 2.74^c^	109.76 ± 0.30^d^	117.30 ± 3.61^cd^	116.99 ± 6.24^cd^	110.15 ± 1.77^d^	158.53 ± 5.44^a^	154.14 ± 0.34^a^	58.35 ± 9.33^f^
Zn (mg/kg)	49.84 ± 2.98^e^	66.07 ± 1.73^b^	73.57 ± 3.98^a^	49.85 ± 1.50^e^	42.79 ± 1.40^f^	56.76 ± 1.07^cd^	57.14 ± 3.24^cd^	58.46 ± 4.08^cd^	54.29 ± 0.02^de^	62.49 ± 1.65^bc^	33.96 ± 1.95^g^
Na (mg/kg)	12.38 ± 0.06^i^	33.10 ± 0.78^e^	39.00 ± 0.63^c^	19.46 ± 1.50^h^	26.67 ± 0.37^f^	47.64 ± 0.69^a^	24.02 ± 0.51^g^	38.06 ± 1.07^c^	35.24 ± 0.62^d^	45.84 ± 0.56^b^	20.41 ± 0.00^h^
Cu (mg/kg)	7.79 ± 0.76^a^	3.34 ± 0.17^c^	1.98 ± 0.35^de^	4.76 ± 0.68^b^	1.33 ± 0.12^e^	1.39 ± 0.12^de^	4.68 ± 0.97^b^	2.25 ± 0.38^cde^	1.80 ± 0.41^de^	2.07 ± 0.22^de^	2.56 ± 0.45^cd^

*All values are mean ± SD*.

*Values followed by different superscript letters in the same row are significantly different (P < 0.05)*.

For the mineral composition of the rice bran samples, calcium was the most abundant mineral component (183.4–383.75 mg/kg), followed by manganese (64.95–154.68 mg/kg) and iron, zinc, and sodium (33.06–82.40, 35.33–70.75, and 12.42–47.16 mg/kg, respectively). While minerals found in the minor contents were potassium, copper, phosphorus, and magnesium (1.29–2.27%, 1.16–7.25 mg/kg, 1.03–1.27%, and 0.45–0.81%, respectively).

#### Relationship Between Rice Bran Variety and Proximate Composition

**Figure 1:**
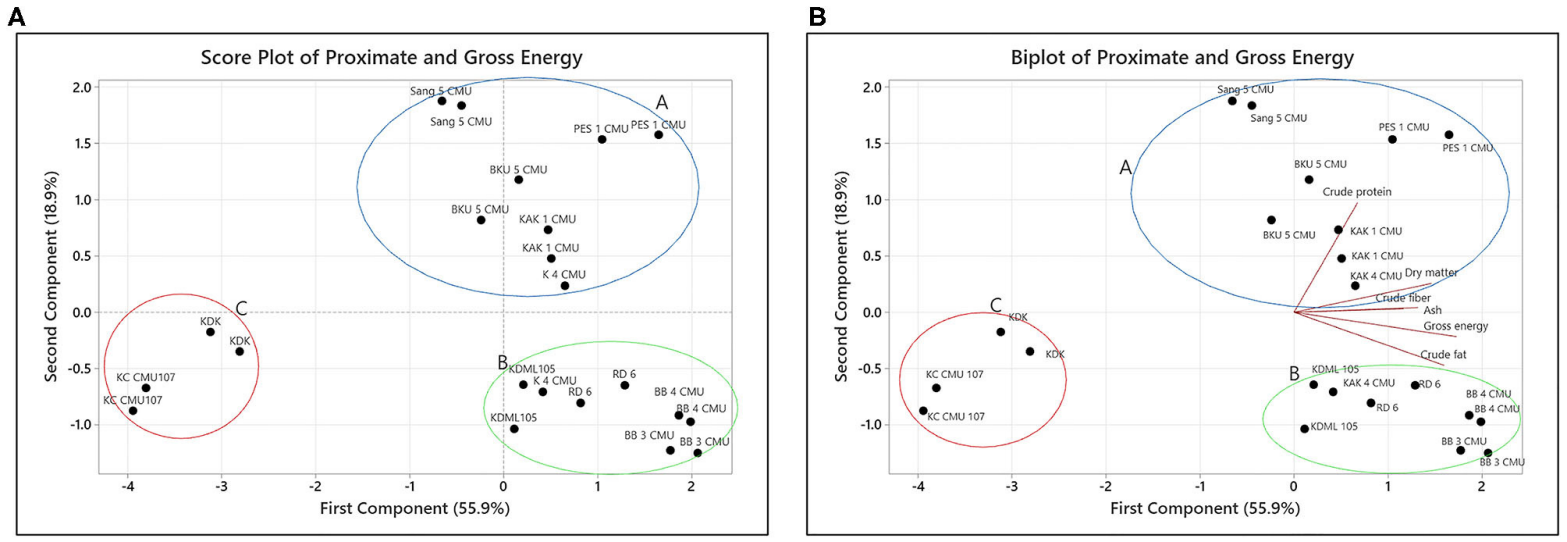
**(A,B)** Principal Component Analysis of rice bran samples.

The relationship between proximate content in the rice bran samples was evaluated using PCA. The first principal component (PC1) accounted for 55.9% and the second principal component (PC2) accounted for 18.9% of the total variation in the results. The eigenvalues for the first four PCs are reported in Table 3. As seen in Figure 1A, three distinct groups were formed. The first group, group A, is located in the first quadrant and contains the varieties Sang 5 CMU, PES 1 CMU, BKU 5 CMU, and KAK 1 CMU but accepted one sample of K 4 CMU. The second group (group B) contains KDML 105, RD 6, BB 3 CMU, and BB 4 CMU and is located in the second quadrant. The third group (group C), containing KDK and KC CMU 107, is located away from others in the third quadrant. In Figure 1B, the PCA biplot shows that the protein content is located in the first quadrant and is close to the major group, A. It can be observed that group B is distantly clustered with the parameters of fat and gross energy. KDK and KC CMU 107 are separated, which might be due to their low nutritional content. The results indicate that each rice variety has different proximate characteristics.

**Table 3:** Eigen analysis of the loadings of the significant principal components (PCs) for the proximate analysis and gross energy content of rice bran varieties.

**Variable**	**PC1**	**PC2**	**PC3**	**PC4**
Dry matter	0.436	0.226	−0.298	0.640
Ash	0.393	0.037	0.679	−0.346
Crude fiber	0.348	0.028	−0.649	−0.660
Crude protein	0.202	0.859	0.128	−0.077
Crude fat	0.477	−0.415	0.110	0.038
Gross energy	0.516	−0.191	0.020	0.163

### γ-Oryzanol Analysis and Determination of Tocopherols

Table 4, shows that the γ-oryzanol content was significantly different among all rice bran samples. Overall, the highest γ-oryzanol content was found in group C, which consisted of KDK and KC CMU 107 (222.34 and 218.76 mg/100 g crude fat, respectively). The lowest γ-oryzanol content was detected in group A (Sang 5 CMU, PES 1 CMU, BKU 5 CMU, and KAK 1) except for K 4 CMU. The highest γ-oryzanol content was found in the sample of purple rice. Tocopherols were also identified, as shown in Table 4. The most abundant tocopherol compound in rice bran samples was α-tocopherol (4.77–17.56 mg/100 g crude fat) followed by γ-tocopherol (4.35–9.19 mg/100 g crude fat). The minor tocopherol compounds found in the sample were β-tocopherol and δ-tocopherol, (0.27–1.16 and 0.11–0.36 mg/100 g crude fat, respectively).

**Table 4:** The γ-oryzanol and tocopherol contents in rice bran samples.

**Group**	**Variety name**	**Compound content (mg/100 g crude fat)**				
		**γ-oryzanol**	**α-tocopherol**	**β-tocopherol**	**γ-tocopherol**	**δ-tocopherol**
A	KAK 1 CMU	175.74 ± 0.25^g^	17.56 ± 0.00^a^	0.87 ± 0.01^ab^	4.44 ± 0.02^c^	0.17 ± 0.00^a^
	BKU 5 CMU	145.16 ± 0.06^i^	15.20 ± 0.01^b^	0.77 ± 0.00^ab^	4.46 ± 0.02^c^	0.18 ± 0.00^a^
	Sang 5 CMU	111.36 ± 0.22^k^	4.77 ± 0.06^de^	0.64 ± 0.00^ab^	6.02 ± 0.04^bc^	0.18 ± 0.00^a^
	PES1 CMU	139.58 ± 0.04^j^	17.02 ± 0.03^ab^	0.72 ± 0.00^ab^	4.35 ± 0.03^c^	0.25 ± 0.00^a^
	K 4 CMU	228.96 ± 0.02^a^	6.39 ± 2.77^e^	0.47 ± 0.95^ab^	8.51 ± 2.92^bc^	0.36 ± 0.25^a^
B	RD 6 CMU	207.79 ± 0.03^f^	9.22 ± 0.06^c^	0.27 ± 0.01^b^	9.19 ± 0.04^a^	0.13 ± 0.00^a^
	KDML105	171.23 ± 0.16^h^	6.62 ± 0.01^d^	0.38 ± 0.00^b^	7.91 ± 0.00^bc^	0.13 ± 0.01^a^
	BB 3 CMU	219.90 ± 0.12^d^	10.37 ± 0.04^c^	0.58 ± 0.00^ab^	6.13 ± 0.02^bc^	0.18 ± 0.00^a^
	BB 4 CMU	220.43 ± 0.09^c^	15.84 ± 0.03^ab^	1.16 ± 0.01^a^	6.78 ± 0.04^b^	0.21 ± 0.01^a^
C	KC CMU 107	218.76 ± 0.13^e^	4.80 ± 0.02^de^	0.62 ± 0.00^ab^	6.28 ± 0.01^c^	0.11 ± 0.00^a^
	KDK	222.34 ± 0.25^b^	5.65 ± 0.04^de^	0.97 ± 0.00^ab^	6.81 ± 0.02^b^	0.22 ± 0.00^a^

*All values are mean ± SD*.

*Values followed by different superscript letters in the same row are significantly different (P < 0.05)*.

### Analysis of Anthocyanins

In the present study, the anthocyanin concentration in rice bran samples was determined by HPLC compared with four standards: cyanin-3,5-diglucoside, cyanidin-3-glucoside, peonidin-3-glucoside, and cyanidin chloride. The results of the experiment are provided in Table 5. The major anthocyanins found in colored rice bran samples was cyanidin-3-glucoside. The highest cyanidin-3-glucoside content was detected in PES 1 CMU (650.55 mg/100 g sample), followed by KAK 1 CMU and BKU 5 CMU (525.72 and 331.10 mg/100 g sample, respectively). The highest peonidin-3-glucoside content was detected in K 4 CMU (100.85 mg/100 g sample), followed by PES 1 CMU and KAK 1 CMU (67.54 and 46.01 mg/100 g sample, respectively). No cyanin-3,5-diglucoside or cyanidin chloride was detected in the rice bran samples in this study.

**Table 5:** The anthocyanins content in rice bran samples.

**Group**	**Variety name**	**C-3-G (mg/100 g sample)**	**P-3-G (mg/100 g sample)**
A	KAK 1 CMU	525.72 ± 1.72^b^	46.01 ± 0.51^c^
	BKU 5 CMU	331.10 ± 1.64^c^	34.26 ± 0.23^d^
	Sang 5 CMU	166.40 ± 0.57^e^	13.09 ± 0.01^g^
	PES1 CMU	650.55 ± 1.65^a^	67.54 ± 0.32^b^
	K 4 CMU	192.87 ± 0.29^d^	100.85 ± 0.29^a^
B	RD 6 CMU	ND	ND
	KDML105	ND	ND
	BB 3 CMU	ND	ND
	BB 4 CMU	ND	ND
C	KC CMU 107	40.61 ± 0.39^f^	15.72 ± 0.13^f^
	KDK	41.86 ± 0.12^f^	23.75 ± 0.68^e^

*All values are mean ± SD; ND, non detectable; Detection limit of each compound: cyanidin-3, 5- diglucoside = 2.26 μg/ml, C-3-G = 19.86 μg/ml, and P-3-G = 2.13 μg/ml*.

*Values followed by different superscript letters in the same row are significantly different (P < 0.05)*.

*C-3-G, cyanidin-3,5-diglucoside; P-3-G = peonidin-3-glucoside*.

### Free Fatty Acid Profile

Analysis of the fatty acid profile of 11 rice brans was accomplished by gas chromatography; the results are shown in Table 6. Thirteen fatty acids components were detected and identified: myristic acid (C14:0), palmitic acid (C16:0), stearic acid (C18:0), arachidic acid (C20:0), eicosadienoic acid (C20:2), heneicosylic acid (C21:0), behenic acid (C22:0), lignoceric acid (C24:0), oleic acid (C18:1*n*9t), oleic acid (C18:1*n*9c), linoleic acid (C18:2*n*6c), γ-linolenic acid (C18:3*n*6) and α-linolenic acid (C18:3*n*3).

**Table 6:** Free fatty acid profile in different varieties of rice bran (g/100 g crude fat).

**Group**	**A**					**B**				**C**	
**Varieties**	**KAK 1 CMU**	**BKU 5 CMU**	**Sang 5 CMU**	**PES 1 CMU**	**K 4 CMU**	**RD 6**	**KDML 105**	**BB 3 CMU**	**BB 4 CMU**	**KC CMU 107**	**KDK**
**Saturated fatty acid (SFA)**											
C14:0	0.52 ± 0.01^c^	0.36 ± 0.02^f^	0.56 ± 0.00^b^	0.60 ± 0.00^a^	0.46 ± 0.01^d^	0.36 ± 0.01^f^	0.27 ± 0.02^g^	0.24 ± 0.00^h^	0.21 ± 0.02^i^	0.38 ± 0.00^e^	0.57 ± 0.00^b^
C16:0	15.14 ± 0.02^h^	14.30 ± 0.02^j^	14.85 ± 0.03^i^	16.47 ± 0.00^e^	19.23 ± 0.01^d^	21.09 ± 0.01^a^	19.67 ± 0.18^c^	15.65 ± 0.02^g^	16.12 ± 0.01^f^	20.15 ± 0.03^b^	19.59 ± 0.00^c^
C18:0	3.30 ± 0.00^a^	2.70 ± 0.00^d^	3.11 ± 0.01^b^	3.03 ± 0.00^c^	2.65 ± 0.00^e^	1.73 ± 0.00^k^	2.17 ± 0.02^h^	2.20 ± 0.00^g^	1.99 ± 0.00^j^	2.02 ± 0.00^i^	2.24 ± 0.00^f^
C20:0	1.58 ± 0.13^ab^	1.33 ± 0.09^bcd^	1.71 ± 0.11^a^	1.57 ± 0.12^ab^	1.51 ± 0.01^ab^	1.49 ± 0.01^ab^	1.06 ± 0.32^d^	1.15 ± 0.09^cd^	1.15 ± 0.10^cd^	1.31 ± 0.07^bcd^	1.40 ± 0.02^bc^
C21:0	0.92 ± 0.02^i^	1.01 ± 0.01^fg^	0.98 ± 0.01^h^	0.99 ± 0.00^gh^	1.15 ± 0.00^d^	1.21 ± 0.00^c^	1.29 ± 0.01^a^	0.99 ± 0.00^gh^	0.90 ± 0.00^i^	1.06 ± 0.00^e^	1.25 ± 0.00^b^
C22:0	0.30 ± 0.02^b^	0.25 ± 0.00^d^	0.30 ± 0.01^b^	0.28 ± 0.00^bc^	0.33 ± 0.00^a^	0.18 ± 0.01^g^	0.25 ± 0.00^de^	0.21 ± 0.02^f^	0.21 ± 0.00^f^	0.23 ± 0.00^ef^	0.22 ± 0.02^f^
C24:0	0.48 ± 0.00^a^	0.45 ± 0.00^b^	0.47 ± 0.02^a^	0.48 ± 0.00^a^	0.16 ± 0.00^f^	0.33 ± 0.01^e^	0.45 ± 0.00^b^	0.38 ± 0.00^d^	0.41 ± 0.00^c^	0.41 ± 0.00^c^	0.40 ± 0.01^c^
**Monounsaturated fatty acid (MUFA)**											
C18:1n9t	0.15 ± 0.02^c^	0.28 ± 0.00^a^	0.09 ± 0.01^fg^	0.10 ± 0.01^ef^	0.12 ± 0.01^de^	0.07 ± 0.00^fg^	0.14 ± 0.02^cd^	0.23 ± 0.03^b^	0.06 ± 0.00^g^	0.14 ± 0.00^cd^	0.16 ± 0.00^c^
C18:1n9c	41.37 ± 0.00^f^	47.51 ± 0.01^a^	41.93 ± 0.05^e^	40.00 ± 0.01^h^	41.36 ± 0.01^f^	37.74 ± 0.02^j^	42.48 ± 0.40^d^	46.76 ± 0.01^b^	45.63 ± 0.01^c^	40.61 ± 0.01^g^	39.46 ± 0.00^i^
**Polyunsaturated fatty acid (PUFA)**											
C18:2n6c	35.69 ± 0.08^ab^	31.14 ± 0.07^e^	35.43 ± 0.01^ab^	35.91 ± 0.12^a^	31.64 ± 0.70^e^	35.31 ± 0.02^b^	31.62 ± 0.29^e^	31.46 ± 0.13^e^	32.69 ± 0.07^d^	33.12 ± 0.07^d^	34.19 ± 0.01^c^
C18:3n6	0.01 ± 0.01^bcd^	0.01 ± 0.00^abcd^	0.02 ± 0.00^a^	0.01 ± 0.01^bcd^	0.01 ± 0.01^bcd^	0.02 ± 0.01^abcd^	0.02 ± 0.00^ab^	0.01 ± 0.03^cd^	0.02 ± 0.00^abc^	0.01 ± 0.00^bcd^	0.01 ± 0.01^cd^
C18:3n3	0.45 ± 0.00^c^	0.56 ± 0.00^a^	0.52 ± 0.01^ab^	0.48 ± 0.00^bc^	0.47 ± 0.00^c^	0.39 ± 0.00^d^	0.48 ± 0.00^bc^	0.53 ± 0.00^ab^	0.55 ± 0.00^a^	0.44 ± 0.00^cd^	0.39 ± 0.00^d^
C20:2	0.10 ± 0.08^cde^	0.14 ± 0.00^bc^	0.08 ± 0.02^fg^	0.08 ± 0.00^efg^	0.07 ± 0.01^g^	0.08 ± 0.00^fg^	0.11 ± 0.00^de^	0.22 ± 0.00^a^	0.06 ± 0.01^g^	0.14 ± 0.00^bc^	0.13 ± 0.00^cd^
SFA	22.23 ± 0.16^e^	20.39 ± 0.09^i^	21.99 ± 0.07^f^	23.43 ± 0.13^d^	25.48 ± 0.0^b^	26.39 ± 0.02^a^	25.16 ± 0.11^c^	20.80 ± 0.08^h^	21.00 ± 0.0^g^	25.56 ± 0.0^b^	25.66 ± 0.0^b^
MUFA	41.52 ± 0.02^f^	47.79 ± 0.0^a^	42.02 ± 0.0^e^	40.09 ± 0.09^h^	41.47 ± 0.03^f^	37.82 ± 0.01^j^	42.62 ± 0.40^d^	46.99 ± 0.02^b^	45.69 ± 0.0^c^	40.75 ± 0.0^g^	39.62 ± 0.01^i^
PUFA	36.25 ± 0.14^ab^	31.85 ± 0.07^e^	36.05 ± 0.0^ab^	36.48 ± 0.11^a^	32.19 ± 0.73^e^	35.80 ± 0.01^b^	32.23 ± 0.29^e^	32.21 ± 0.10^e^	33.09 ± 0.08^d^	33.70 ± 0.07^d^	34.72 ± 0.02^c^

*All values are mean ± SD; SFA, saturated fatty acids; MUFA, monounsaturated fatty acids; PUFA, polyunsaturated fatty acids*.

*Values followed by different superscript letters in the same row are significantly different (P < 0.05)*.

The non-glutinous rice sample, BKU 5 CMU, showed the highest content of oleic acid (47.51 g/100 g crude fat) followed by BB 3 CMU (46.76 g/100 g crude fat) and BB 4 CMU (45.63 g/100 g crude fat), while the lowest was observed in the group of glutinous rice, including RD 6 (37.74 g/100 g crude fat) and KDK (39.46 g/100 g crude fat). The highest linoleic acid content was detected in the black glutinous rice samples, PES 1 CMU (35.91 g/100 g crude fat) followed by KAK 1 CMU (35.68 g/100 g crude fat) and Sang 5 CMU (35.43 g/100 g crude fat), and the lowest contents were exhibited in the non-glutinous rice samples. The palmitic acid content varied among rice bran samples.

### Phenolic Compounds

The content of polyphenolic compounds in different varieties of rice bran samples is summarized in Table 7. Sixteen compounds were identified by comparison of their mass spectra and retention times of 17 standards available in the laboratory. The phenolic compounds with a high content in the rice bran samples were phytic acid (0.26–8.65 mg/100 g sample), quercetin (0.17–1.44 mg/100 g sample), and chlorogenic acid (0.5–10.93 mg/100 g sample). Ferulic acid, epigallocatechin gallate, *p*-coumaric acid, *o*-coumaric acid, and naringin were also detected in all rice bran samples.

**Table 7:** Phenolic contents in different varieties of rice bran (mg/100g sample).

**Group**	**A**					**B**				**C**	
**Varieties**	**KAK 1 CMU**	**BKU 5 CMU**	**Sang 5 CMU**	**PES 1 CMU**	**K 4 CMU**	**KDML 105**	**BB 3 CMU**	**BB 4 CMU**	**RD 6**	**KC CMU 107**	**KDK**
Gallic acid	ND	ND	ND	ND	ND	0.09 ± 0.01^c^	0.14 ± 0.00^b^	0.15 ± 0.00^a^	0.13 ± 0.00^b^	ND	ND
Caffeic acid	0.15 ± 0.00^e^	0.40 ± 0.02^a^	0.16 ± 0.01^e^	0.26 ± 0.00^c^	ND	0.17 ± 0.01^e^	0.30 ± 0.01^b^	0.31 ± 0.00^b^	0.19 ± 0.00^d^	0.12 ± 0.00^f^	0.09 ± 0.00^g^
Catechin	ND	ND	ND	ND	ND	ND	ND	ND	ND	0.04 ± 0.00^a^	ND
Epicatechin	0.13 ± 0.00^c^	0.09 ± 0.00^d^	0.22 ± 0.05^b^	0.33 ± 0.01^a^	ND	ND	ND	0.09 ± 0.00^d^	ND	ND	0.08 ± 0.00^d^
Epigallocatechin gallate	0.50 ± 0.00^e^	0.47 ± 0.01^ef^	0.42 ± 0.03^ef^	0.39 ± 0.00^fg^	0.68 ± 0.02^d^	0.42 ± 0.09^ef^	1.34 ± 0.06^a^	0.96 ± 0.04^c^	1.21 ± 0.02^b^	0.32 ± 0.00^gh^	0.28 ± 0.01^h^
*p*-coumaric acid	0.33 ± 0.01^e^	0.46 ± 0.01^c^	0.23 ± 0.04^f^	0.26 ± 0.00^f^	0.44 ± 0.01^c^	0.36 ± 0.01^de^	1.15 ± 0.07^a^	0.87 ± 0.01^b^	0.41 ± 0.02^cd^	0.22 ± 0.01^f^	0.15 ± 0.00^g^
*o*-coumaric acid	0.33 ± 0.02^cd^	0.05 ± 0.03^ab^	0.55 ± 0.03^a^	0.39 ± 0.01^bc^	0.25 ± 0.02^de^	0.57 ± 0.04^a^	0.61 ± 0.17^a^	0.57 ± 0.01^a^	0.51 ± 0.00^ab^	0.15 ± 0.00^e^	0.18 ± 0.04^e^
Naringin	0.22 ± 0.00^de^	0.80 ± 0.02^a^	0.58 ± 0.08^b^	0.17 ± 0.05^de^	0.22 ± 0.04^de^	0.16 ± 0.00^de^	0.87 ± 0.05^a^	0.44 ± 0.04^c^	0.25 ± 0.00^d^	0.19 ± 0.01^de^	0.13 ± 0.03^e^
Rosmarinic acid	0.06 ± 0.01^cd^	ND	ND	0.10 ± 0.02^b^	0.14 ± 0.00^a^	0.07 ± 0.00^c^	ND	0.13 ± 0.00^a^	ND	0.06 ± 0.00^cd^	0.05 ± 0.00^d^
Quercetin	1.21 ± 0.01^c^	1.15 ± 0.01^d^	1.27 ± 0.01^b^	1.44 ± 0.00^a^	1.26 ± 0.02^b^	0.27 ± 0.04^f^	0.26 ± 0.00^f^	0.24 ± 0.01^f^	0.17 ± 0.00^g^	1.22 ± 0.01^c^	0.81 ± 0.00^e^
Naringenin	ND	ND	ND	ND	ND	ND	ND	ND	ND	ND	ND
Rutin	0.07 ± 0.00^d^	0.09 ± 0.00^c^	ND	ND	0.23 ± 0.00^a^	ND	ND	ND	ND	0.12 ± 0.00^b^	0.09 ± 0.00^c^
Phytic acid	5.36 ± 0.05^b^	ND	ND	1.19 ± 0.00^f^	1.57 ± 0.01^e^	1.14 ± 0.04^f^	3.29 ± 0.05^c^	8.65 ± 0.12^a^	0.26 ± 0.14^d^	0.72 ± 0.05^g^	0.59 ± 0.03^g^
Ferulic acid	0.23 ± 0.01^d^	0.28 ± 0.00^b^	0.22 ± 0.00^de^	0.24 ± 0.01^c^	0.20 ± 0.01^f^	0.17 ± 0.01^g^	0.18 ± 0.00^g^	0.17 ± 0.01^g^	0.21 ± 0.01^ef^	0.33 ± 0.00^a^	0.13 ± 0.00^h^
Chlorogenic acid	0.78 ± 0.02^c^	0.84 ± 0.04^bc^	0.79 ± 0.01^c^	0.93 ± 0.02^a^	0.86 ± 0.01^b^	0.61 ± 0.01^d^	0.87 ± 0.05^ab^	0.65 ± 0.04^d^	0.84 ± 0.00^bc^	0.64 ± 0.03^d^	0.51 ± 0.02^e^
Kaempferol	0.07 ± 0.00^d^	0.07 ± 0.01^d^	0.07 ± 0.01^d^	0.11 ± 0.04^c^	ND	ND	0.26 ± 0.01^a^	0.16 ± 0.00^b^	ND	ND	ND
Hydroxybenzoic acid	0.27 ± 0.00^d^	ND	0.48 ± 0.03^b^	0.37 ± 0.02^c^	0.30 ± 0.03^d^	0.29 ± 0.07^d^	ND	ND	ND	0.59 ± 0.02^a^	0.30 ± 0.01^d^

*All values are mean ± SD; ND, non detectable; Detection limit of each compound: Gallic acid = 0.12 μg/ml, Caffeic acid = 0.06 μg/ml, Catechin = 0.30 μg/ml Epicatechin = 3.01 μg/ml, Rosmarinic acid = 0.88 μg/ml, Naringenin = 0.56 μg/ml, Rutin = 1.07 μg/ml, Phytic acid = 14.68 μg/ml, Kaempferol = 0.92 μg/ml, Hydroxybenzoic acid = 0.95 μg/ml*.

*Values followed by different superscript letters in the same row are significantly different (P < 0.05)*.

## Discussion

Rice bran is a by-product of rice kernel dry milling that mostly consists of the germ, pericarp, aleurone, and sub-aleurone layers. It is an excellent natural source of protein (14–16%), fat (12–23%), crude fiber (8–10%), carbohydrates, and other bioactive compounds, all of which have well-known health benefits (13). Different rice varieties have been shown to have varying mineral contents (16, 31). Minerals, both macro, and micro such as Na, K, Mg, Ca, Zn, Fe, Cu, and Mn are also recognized as important and significant in biological systems because of their possible role in regulating body activities (12).

The appearance of food, particularly plant matrices, is a key factor in consumer decision-making. To determine the relationship between biological parameters, the preliminary report of Wisetkomolmat et al. (32) used multivariate PCA to analyze the variables of the bioactive compounds in Thai local plants. This method is the most fundamental technique used in chemometrics; it can minimize the data dimension and visually cluster variants using principal components and has been applied in various fields of biology and chemistry. In this study, PCA was used to group rice into three main groups with different characteristics, described as follows:

From the PCA results, it can be assumed that group A has a high protein and fiber content. The results for the protein and fat content are in the same ranges as previous observations of the same varieties (KDML 105 and RD 6) reported by Moongngarm et al. (33). In their study, Amagliani et al. (16) noticed that rice bran had a protein content that was two-fold higher than that of other seed parts. Protein in rice bran is predominantly concentrated in its outer section, according to Schramm et al. (34) as indicated by the fact that the protein content of bran samples can be decreased with increasing milling time. Moreover, rice's protein content also has a significant impact on its structural, functional, and nutritional qualities (16). The differences in protein content between rice varieties are due to the different mechanisms for accumulating protein in the grains. The results from our study support prior research showing that grain protein levels are significantly affected by rice variety (11). For other components, cellulose, hemicellulose, lignin, pectin, and gums are the principal forms of fiber found in rice (some hemicelluloses and storage polysaccharides) (16). Rice digestibility is affected by the crude fiber level, with a high crude fiber content in rice lowering its digestion (35). Ash content plays an important role in reflecting the mineral elements of a food sample. The experimental findings for the content of ash and protein were the same as those of Amagliani et al. (16), which were mainly found in the bran layer rather than the others.

Natural antioxidants in food are gaining popularity due to their possible function in the prevention of oxidative stress-related illnesses. Rice bran is reported to contain significant levels of natural antioxidants in varying amounts and proportions depending on the variety (36). Anthocyanins are found in nature as anthocyanidin aglycones and anthocyanin glycosides, but they can also be conjugated with aliphatic or phenolic acids. The inclusion of sugars and phenolic acids aids the stability of anthocyanins (21, 37). A high level of anthocyanins was detected in group A which is a group of purple rice. From this point of view, the rice bran varieties in group A were discovered to mainly contained protein and fiber composition. This group of rice bran also had a high content of anthocyanins. As a result, consumers could choose to consume this group of rice in order to get antioxidants and a good source of protein and dietary fiber.

For group B, the PCA results indicate that the second group of rice bran samples have a high fat and a high gross energy level. Examples of rice bran in group B are all non-colored rice. Rice fat is high in linoleic and other important fatty acids, and it is low in cholesterol (35). Although lipid is a minor component of rice, it has important nutritional, sensory, and functional qualities (16). Food energy is a measure that represents the amount of energy that can be gained from food through cellular respiration. The differences in outcomes could be attributed to variances in the cultivars utilized, the soil type, or the environment (38). There were some differences presented in fatty acid contents. In addition, the non-glutinous rice samples had the highest content of oleic acid. The highest content of linoleic acid was detected in the black glutinous rice samples. The variety, planting region, climate circumstances, and post treatment process can all play a role in the fatty acid content (10). Oleic, linoleic, and palmitic acids were the major fatty acids in all rice bran samples. The content of unsaturated fatty acids was higher than that of saturated fatty acids. This is relevant to the outcome of the study of Verma and Srivastav (35) which found oleic, linoleic, and palmitic acids are important fatty acids in all aromatic and non-aromatic rice accessions, whereas linolenic, myristic, and stearic acids are minor components. The fatty acid profiles determined in this study are also in accordance with the result of Punia et al. (15) who found that rice bran oil includes a variety of fatty acids, including 47% monounsaturated, 33% polyunsaturated, and 20% saturated fatty acid. Rice bran's health-promoting qualities come from unsaturated fatty acids which act as antioxidants and anti-cancer agents by stimulating the formation of compounds that protect cells from peroxides (39). This group of rice bran samples (KDML 105, RD 6, BB 3 CMU, and BB 4 CMU) could be used as a raw material for oil bran oil production.

By displaying the samples using the PCA technique, it was discovered that the sample in group C had low fat and energy values but contained high amounts of functional active components, particularly γ-oryzanol, in contrast to group A which had the lowest concentration of γ-oryzanol. Rice bran oil contains bioactive components including tocopherols and tocotrienols (vitamin E) and γ-oryzanol (10, 17), a combination of trans-ferulic acid esters of triterpene alcohols and sterols (15). Several investigations have suggested that these substances have antioxidant, hypocholesterolemic, and anti-diabetic properties (16, 21). However, the results for γ-oryzanol and tocopherol, both α- and γ-tocopherol were slightly higher than the previous observations in the same varieties of non-colored rice bran (KDML 105 and RD 6) reported by Moongngarm et al. (33) and also the results from a report by Huang and Lai (40) on Thai colored rice bran. However, our result was in contrast with the report of Heinemann et al. (41) which found a positive relationship between α-tocopherol and γ-oryzanol content in colored rice bran samples. There was no positive relationship between α- and γ-homologs that could be explained by the fact that these two molecules have independent biosynthesis pathways (42). Iqbal et al. (18) suggested that tocols and oryzanols are the main antioxidants present in rice bran. Oryzanols have a nearly tenfold greater antioxidant activity than tocopherols. As a result, rice bran, which contains a unique complex of oryzanols and tocols, may be a useful source of chemicals for inhibiting lipid peroxidation. Because the structure of γ-oryzanol components is similar to that of cholesterol, an important component in reducing oxidation stress and maintaining cell functionality, structural relationship theory suggests that the γ-oryzanol components may have a greater ability to associate with cholesterol in the small droplets of an emulsion and become more efficient in protecting cholesterol (43).

Phenolic substances are secondary metabolites found in plants that can scavenge free radicals, reducing oxidative stress and protecting biological macromolecules from possible risk (44). Phenolic chemicals' anti-oxidant characteristics are responsible for the prevention of chronic diseases such as obesity, diabetes, atherosclerosis, cancer, and cardiovascular disorders (12, 44). The composition of the phenolic compounds was slightly different among rice bran samples, colored and non-colored. Rice bran contains phenolics in three different forms: soluble free, soluble conjugated, and insoluble bound. The antioxidant capacity of bound phenolics is significantly higher than that of free or soluble conjugated forms with ferulic acid being the major phenolic compound present (43). In contrast. we found phytic acid as the most abundant phenolic compound; this may be due to ferulic acid being esterified to cell wall components in rice and so becoming part of the dietary fiber, where it exists in an insoluble bound form (45). The results show that consumers could choose this group of rice bran varieties (KDK and KC CMU 107) as part of a healthy diet with a low fat content but high active ingredient content.

The results stated above indicate that each type of rice bran has different characteristics but they can be grouped using the multivariate PCA technique. The type, appearance, and bioactive content of rice bran are also influenced by the geographical origin and genetic variation among rice species (46). There is growing evidence that increasing one's intake of foods relatively rich in natural antioxidants, such as plant polyphenols, and vitamin E, can reduce the risk of chronic and degenerative diseases (18). The research outcome could be utilized as a guideline for selecting rice bran that is suitable for the needs of the industrial sector and to encourage the consumer to select Thai rice bran that have potential and are acceptable for daily nutrients requirements. However, future study should focus on the activity of these active compounds in order to have a better understanding.

## Conclusion

Due to an increase in awareness of nutrition and health, attention has turned to improving functional food products containing bioactive components. This research was undertaken to describe the nutrient composition and bioactive compound profile of 11 Thai rice brans with special qualities. Rice bran includes a variety of nutrients, such as fiber, minerals, and vitamins, as well as health-promoting bioactive phytochemicals such as γ-oryzanol, tocopherols, anthocyanins, and phenolic compounds. Both colored and non-colored rice bran varieties have a unique characteristic profile of bioactive components. Statistical analysis was performed to correlate the results obtained from PCA in order to categorize the samples by nutritional characteristics into three main groups: group A with a high protein and fiber content, group B with a high fat and gross energy content, and group C with low fat and low energy values but high amounts of functional, active components, particularly γ-oryzanol. Only one sample of colored rice bran was found to contain anthocyanins. Oleic, linoleic, and palmitic acids were the most common free fatty acids detected in rice bran samples. Future research and applications of each rice bran component in the development of functional foods or related items as value-added ingredients will benefit from the findings of this study.

## Data Availability Statement

The original contributions presented in the study are included in the article/supplementary material, further inquiries can be directed to the corresponding authors.

## Author Contributions

CP-u-T and KS: conceptualization. JW, CA, and AS: methodology and investigation. MS-a and WR: validation. JW and CA: data curation. WR and KS: project administration. WR, KS, and CP-u-T: resources. JW: writing—original draft. CA, WR, CP-u-T, and KS: writing—review and editing. All authors have read and agreed to the published version of the manuscript.

## Funding

This research project was supported by Fundamental Fund 2022, Chiang Mai University and in part by the Office of Research Administration, Chiang Mai University.

## Conflict of Interest

The authors declare that the research was conducted in the absence of any commercial or financial relationships that could be construed as a potential conflict of interest.

## Publisher's Note

All claims expressed in this article are solely those of the authors and do not necessarily represent those of their affiliated organizations, or those of the publisher, the editors and the reviewers. Any product that may be evaluated in this article, or claim that may be made by its manufacturer, is not guaranteed or endorsed by the publisher.
